# Deformation-driven catalysis of nanocrystallization in amorphous Al alloys

**DOI:** 10.3762/bjnano.7.134

**Published:** 2016-10-11

**Authors:** Rainer J Hebert, John H Perepezko, Harald Rösner, Gerhard Wilde

**Affiliations:** 1 Department of Materials Science and Engineering, University of Connecticut, Storrs, 97 N Eagleville Rd., Storrs, CT 06269, USA; 2 Department of Materials Science and Engineering, University of Wisconsin-Madison, 1509 University Avenue, Madison, Wisconsin, 53706, USA; 3 Institut für Materialphysik, Universität Münster, Wilhelm-Klemm Str. 10, 48149 Münster, Germany

**Keywords:** amorphous alloy, annealing, cold-rolling, nanocrystal, shear-band

## Abstract

Nanocrystals develop in amorphous alloys usually during annealing treatments with growth- or nucleation-controlled mechanisms. An alternative processing route is intense deformation and nanocrystals have been shown to develop in shear bands during the deformation process. Some controversy surrounded the idea of adiabatic heating in shear bands during their genesis, but specific experiments have revealed that the formation of nanocrystals in shear bands has to be related to localized deformation rather than thermal effects. A much less debated issue has been the spatial distribution of deformation in the amorphous alloys during intense deformation. The current work examines the hypothesis that intense deformation affects the regions outside shear bands and even promotes nanocrystal formation in those regions upon annealing. Melt-spun amorphous Al_88_Y_7_Fe_5_ alloy was intensely cold rolled. Microcalorimeter measurements at 60 °C indicated a slight but observable growth of nanocrystals in shear bands over the annealing time of 10 days. When the cold-rolled samples were annealed at 210 °C for one hour, transmission electron images did not show any nanocrystals for as-spun ribbons, but nanocrystals developed outside shear bands for the cold rolled samples. X-ray analysis indicated an increase in intensity of the Al peaks following the 210 °C annealing while the as-spun sample remained “X-ray amorphous”. These experimental observations strongly suggest that cold rolling affects regions (i.e., spatial heterogeneities) outside shear bands and stimulates the formation of nanocrystals during annealing treatments at temperatures well below the crystallization temperature of undeformed ribbons.

## Findings

Crystallization reactions in metallic glasses have been extensively studied due to the beneficial effect of nanocrystal dispersions on mechanical [1–4] and magnetic properties [5–8], but also as experimental case studies for nucleation and growth theories [9–11]. The devitrification of metallic glasses is commonly considered as a thermally activated process, but some glassy alloys crystallize during intense deformation at temperatures well below the glass transition temperature. Examples of deformation-induced crystallization reactions include Fe-based amorphous alloys [12–16], amorphous Ni–P alloy [17], bulk amorphous Zr and Cu–Zr alloys, and amorphous Al alloys [18–22]. The deformation techniques used so far include bending [23], ball milling [14–16,24], cold rolling [12,25–32], high-pressure torsion straining [18,33], nanoindentation [34], and uniaxial compression [35]. The results of intense deformation have demonstrated nanocrystals with number densities of up to 10^22^ m^−3^ and average sizes of less than 10 nm, but the origin of the deformation-driven nanocrystallization remains an area of active research. Intense deformation in metallic glasses occurs in shear bands at stress levels of more than about 30% of the shear modulus and at temperatures of below approximately 0.7·*T*_g_ [36]. If intense deformation can induce nanocrystals in some amorphous alloys and the intense deformation occurs within shear bands, a key question then is how the nanocrystal formation is related to the shear bands. Literature results clearly point toward the formation of nanocrystals within shear bands [37] and the shear band crystallization can be motivated with enhanced mobility within the shear bands [37] and the localization of atomic motion within shear bands [38]. Comparatively little is known, however, about the effect of intense deformation on the amorphous phase surrounding the shear bands. The common conception is that the amorphous matrix remains unchanged during deformation [3]. On the other hand, Gupta concluded based on Mössbauer spectroscopy of an Fe-based amorphous alloy after cold rolling that the deformation-induced atomic rearrangements exist throughout the entire sample and not only in shear bands [39]. This letter yields further evidence that intense deformation does indeed affect the regions between shear bands and even promotes crystallization in non-shear band regions.

**Figure 1:**
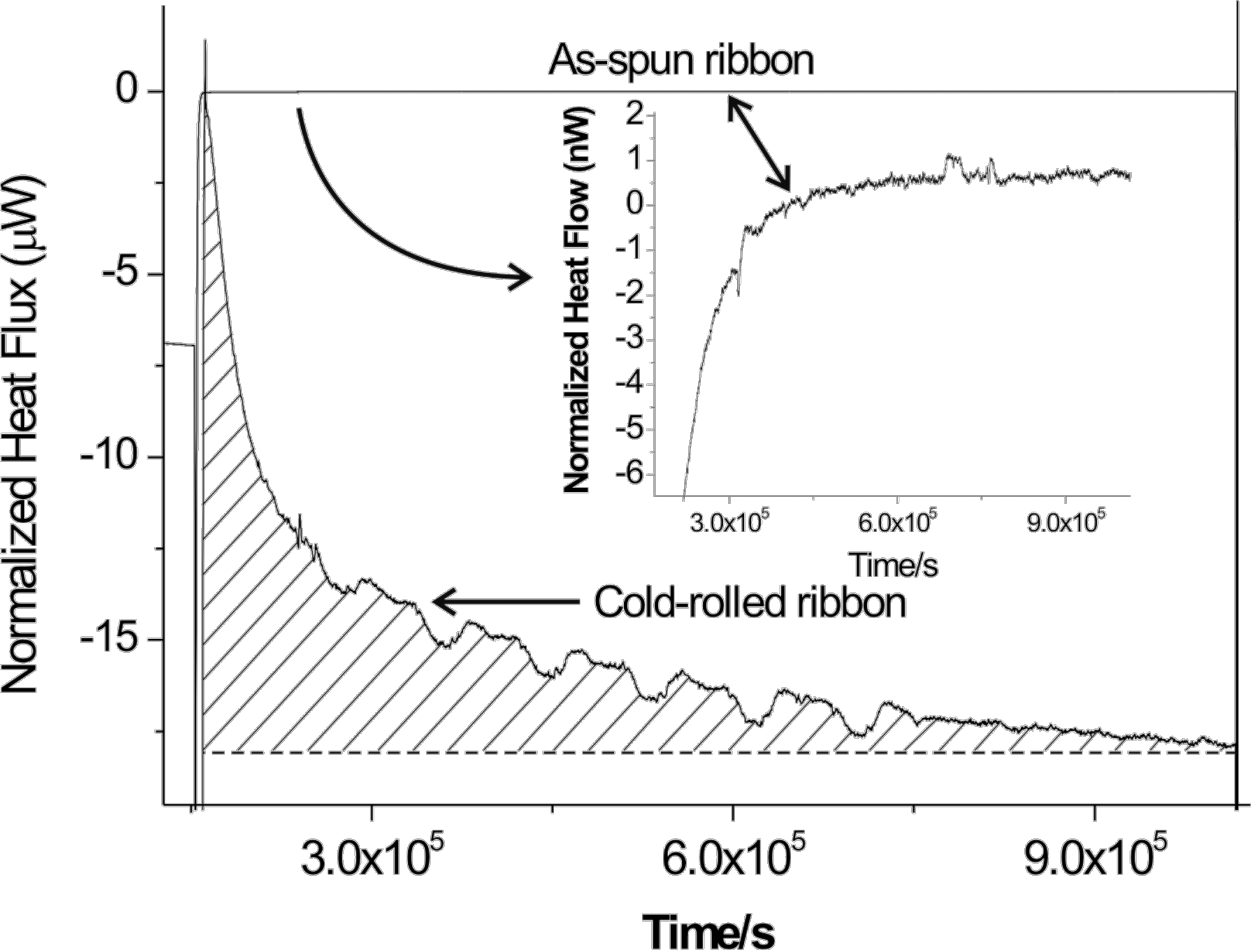
Isothermal microcalorimeter trace at 60 °C for as-spun and cold-rolled ribbons. The inset shows the trace of the as-spun ribbon at a scale of nanowatts.

**Figure 2:**
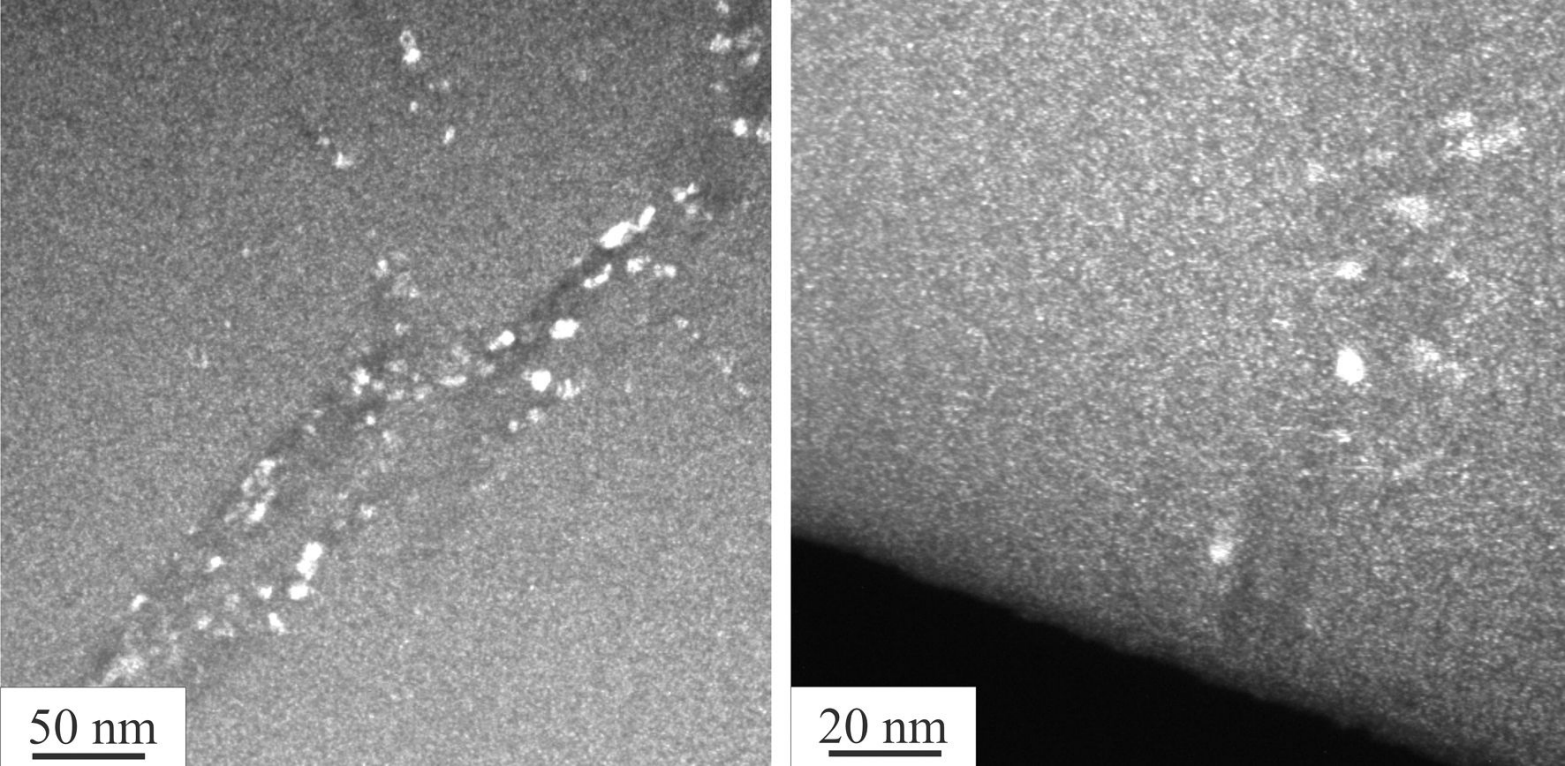
Dark field TEM analysis of cold-rolled (left) and cold-rolled and annealed samples. The sample was annealed at 60 °C for 10 days (right).

**Figure 3:**
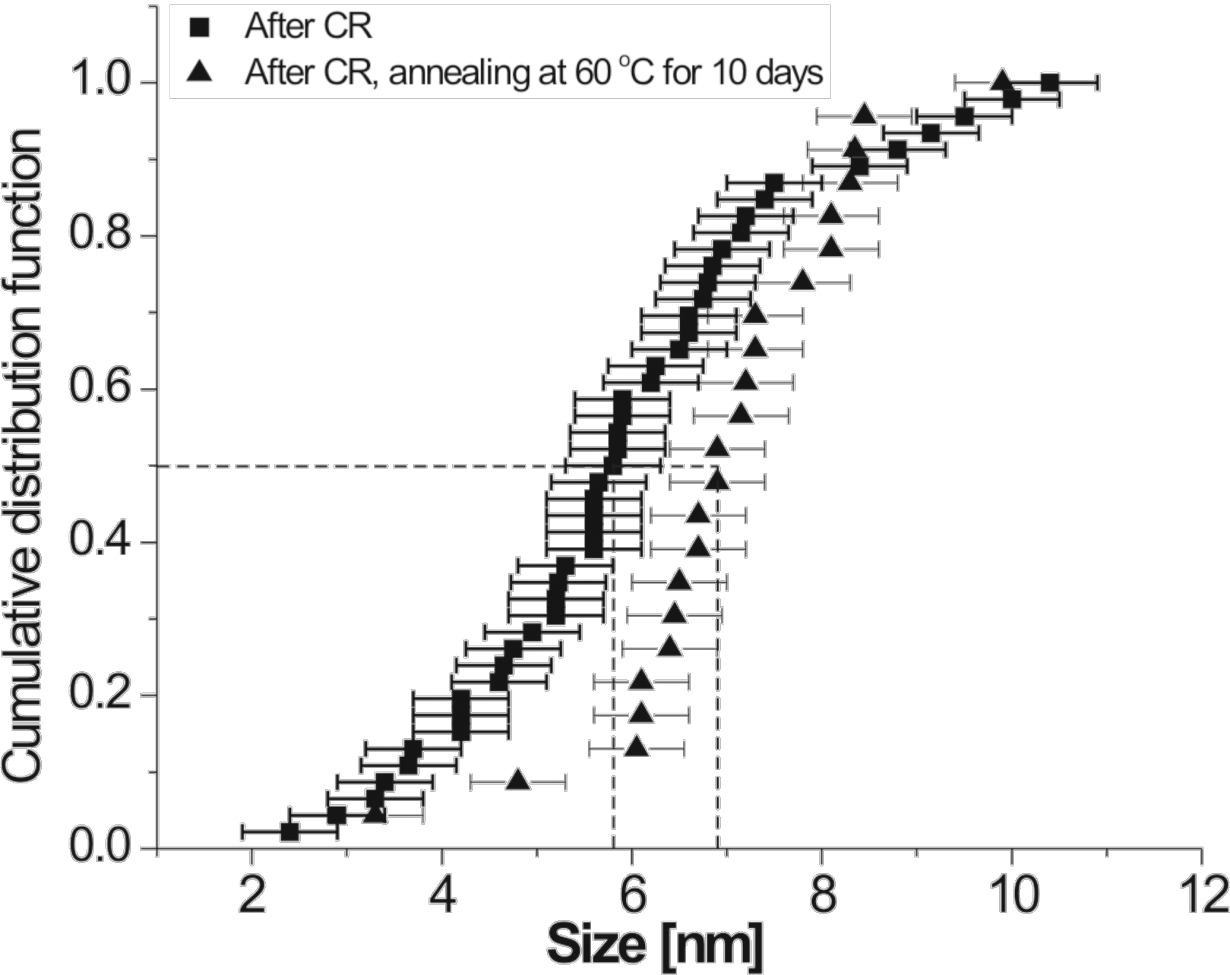
The cumulative size distribution for Al nanocrystals inside shear bands. Square markers represent measurements on samples that were only cold-rolled. Triangular markers represent samples that were cold-rolled and annealed at 60 °C for 10 days.

Melt-spun amorphous Al_88_Y_7_Fe_5_ alloy was annealed in a TAM microcalorimeter (TA Instruments) at 60 °C, i.e., about 200 K below the glass-transition temperature [40]. The isothermal microcalorimeter trace of the as-spun ribbon shown in Figure 1 demonstrates that during the annealing time of 10 days the heat flux reaches a plateau with fluctuations on the order of approximately 7 nW/g. The trace for the as-spun ribbon represents the instrument baseline. If the same amorphous Al_88_Y_7_Fe_5_ alloy is cold-rolled at room temperature to ε_true_ = −3 at a strain rate of 0.1 s^−1^, the same microcalorimeter measurement reveals a clear exothermic heat release as also shown in Figure 1. The total heat release (hatched area in Figure 1) amounts to about 2 J/g. The dark-field (DF) TEM analysis of both samples, the cold-rolled sample and the cold-rolled sample after annealing in the microcalorimeter, shows clear evidence of nanocrystals in shear bands of both samples (Figure 2). The TEM images shown in this work were obtained from samples prepared by grinding, polishing, and electrolytic thinning with dilute perchloric acid. The nanocrystals in the partially devitrified Al_88_Y_7_Fe_5_ alloy have been identified as Al nanocrystals [40]. The size distribution analysis of the Al nanocrystals inside the shear bands of the rolled sample before and after the annealing, which is depicted in Figure 3 reveals a small but definite shift in the size distribution after the annealing. The size of the nanocrystals could be determined with an uncertainty of approximately 1 nm. The error bars in Figure 3 show that despite the measurement uncertainty, a trend of nanocrystal growth behavior is evident. The size measurement for the size distribution in Figure 3 refers to the longest projected diameter.

**Figure 4:**
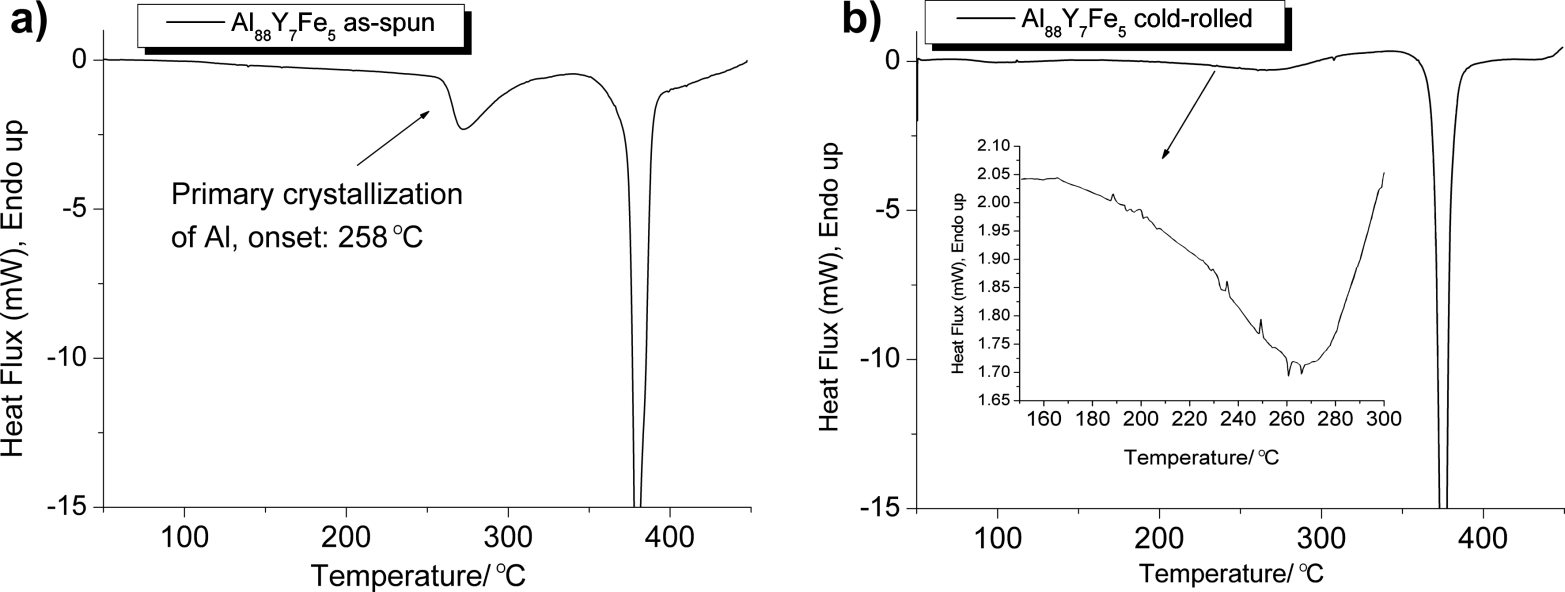
(a) DSC trace for an as-spun Al_88_Y_7_Fe_5_ ribbon sample. (b) DSC trace for the cold-rolled Al_88_Y_7_Fe_5_ ribbon sample. The inset shows the primary crystallization range after cold rolling. The heating rate was 20 K/min for both measurements.

**Figure 5:**
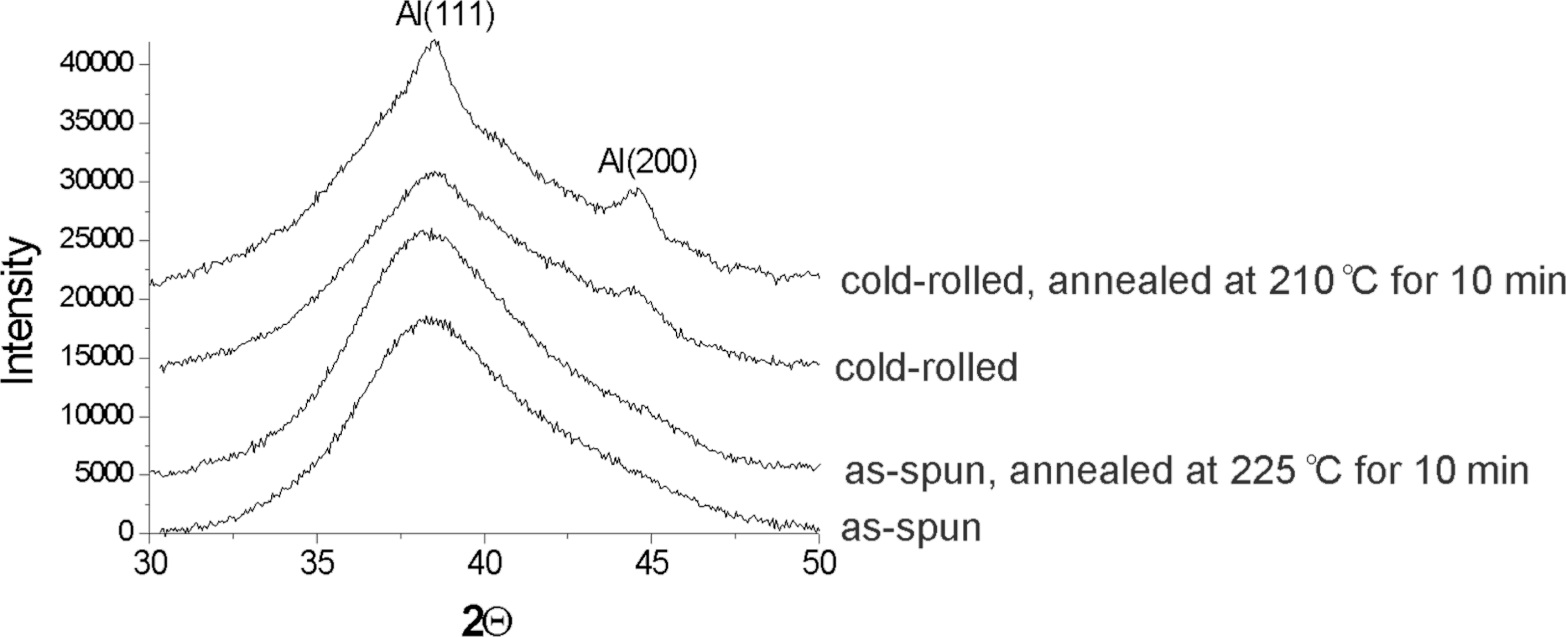
XRD results showing an increase in intensity of the Al peaks after annealing of a cold-rolled sample.

**Figure 6:**
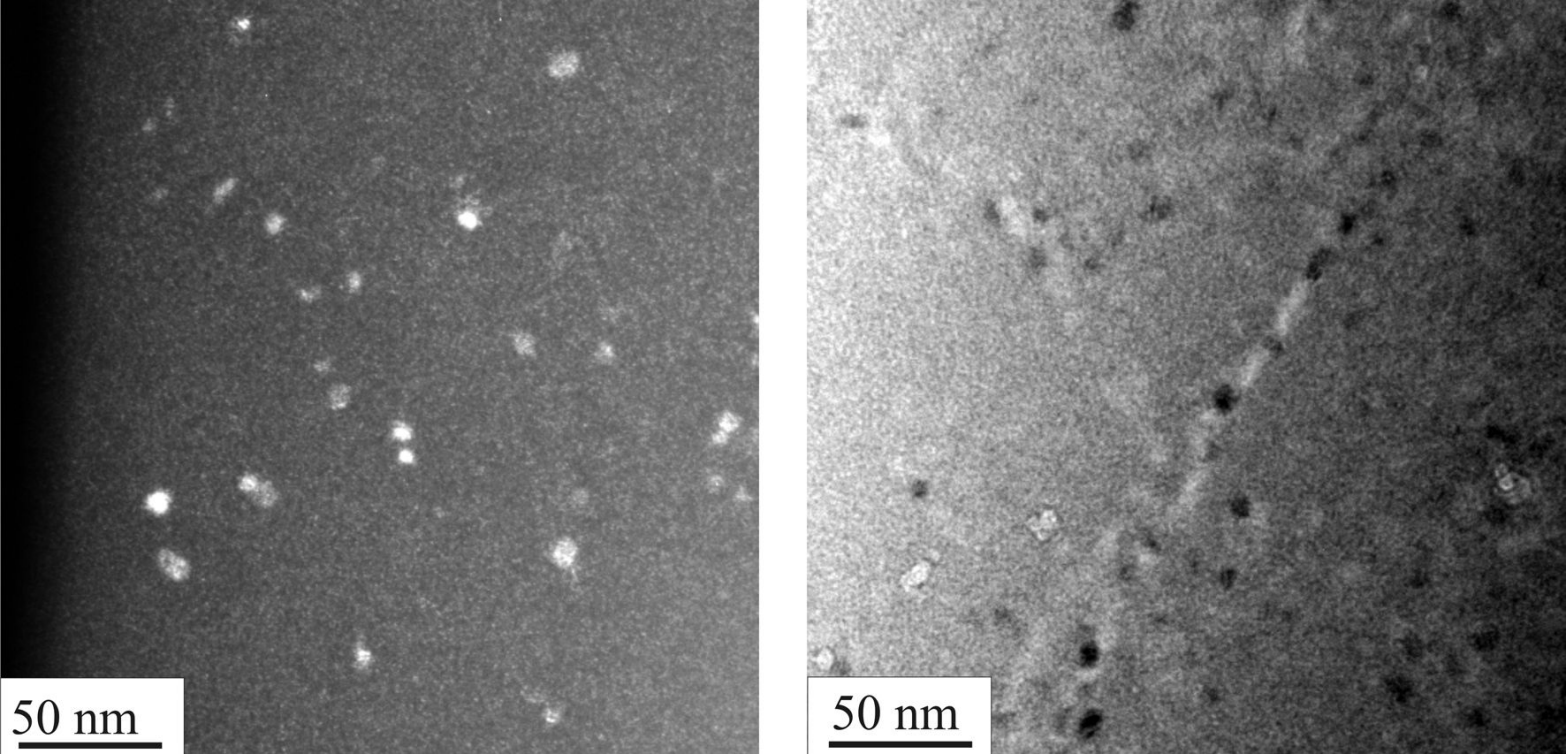
DF TEM (left) and BF TEM (right) after 210 °C anneal of cold-rolled sample.

The difference between the as-spun and the cold-rolled samples after annealing at 60 °C seems at first glance to be limited to the formation of shear bands and nanocrystals inside the shear bands of the deformed sample. Possible atomic rearrangements resulting from the intense deformation outside shear bands are difficult to detect with electron microscopy. But if it is hypothesized that the deformation locally rearranges atoms to promote the formation of nanocrystals, then an adequate probe for the hypothesis should be additional annealing treatments. If the intensely deformed samples are annealed at temperatures much below the crystallization onset temperature of the as-spun material, deformation outside of shear bands might trigger the formation of nanocrystals during the annealing treatment. The continuous heating differential scanning calorimetry (DSC) trace of a cold-rolled Al_88_Y_7_Fe_5_ ribbon that is depicted in Figure 4 shows that the onset temperature for the primary crystallization reaction is approximately 175 °C for the cold-rolled sample. By comparison, the undeformed amorphous Al_88_Y_7_Fe_5_ alloy reveals a primary crystallization onset temperature of approximately 260 °C. Cold-rolled Al_88_Y_7_Fe_5_ ribbons were annealed at 210 °C for 10 min and were subsequently analyzed with X-ray diffraction (XRD) and with transmission electron microscopy. The comparison between the different XRD curves depicted in Figure 5 shows an increase in the intensity of the Al peaks after annealing of the cold-rolled sample. An as-spun sample, on the other hand, remained X-ray amorphous after annealing for 10 min at 225 °C. The dark-field TEM image in Figure 6 shows that nanocrystals have developed homogeneously throughout the deformed sample rather than in localized regions. Shear bands are not detected in the dark-field TEM images after the annealing. With shear band distances of the order of micrometers [41] and good contrast between shear bands and matrix in Al-based amorphous alloys [42], it can therefore be concluded that the nanocrystals in Figure 6 that are observed after the annealing at 210 °C developed in the matrix and not in shear bands.

The combination of HRTEM, DF-TEM, XRD, and DSC results provides strong evidence that cold-rolling does promote crystallization outside shear bands at annealing temperatures that are insufficient to induce crystallization for undeformed samples. This cold-rolling enabled crystallization upon annealing in regions outside shear bands could be perceived of as a result of heating due to the temperature spikes in the shear bands. The localized heating in the shear band would spread into the adjacent matrix and promote crystallization. The question of adiabatic heating in shear bands during deformation has been discussed in the literature quite extensively. Evidence was provided for temperature spikes with shear band formation [43], but models were also developed that demonstrated stick–slip of shear bands and “cold” temperatures in the shear bands [44]. The findings on temperature behavior in shear bands of metallic glasses over the last years were summarized by Greer, Cheng, and Ma [45]. One of the major findings of recent years was that heating occurs as a result of shear-banding but not as a reason for the formation of shear bands. It was also found that the heating in shear bands is not truly adiabatic, but extends beyond the regions that can be identified as shear bands in images after deformation [45]. Additional evidence in the current work for a limited temperature rise at shear bands comes from the TEM images of deformed samples: The regions adjacent to the shear bands are just amorphous without any evidence for a gradual transition from a nanocrystal-populated zone toward an amorphous zone that would be expected if the shear bands were heat sources. If shear band heating cannot account for the observed annealing response of cold-rolled Al_88_Y_7_Fe_5_ ribbons, a plausible conclusion is that regions outside shear bands are affected by the cold rolling and induce atomic rearrangements that promote nanocrystal formation during subsequent annealing. This conclusion resonates with earlier literature results. Inoue and coworkers, for example, explained the changes in superconducting properties of their Nb_50_Zr_35_Si_15_ metallic glass after cold rolling with deformation-induced structural rearrangements that occurred not only in shear bands, but also in the region between the shear bands [46]. Argon [47] and Steif [48] demonstrated that shear localization was preceded by homogeneous plastic flow. Cahn and co-workers concluded based on density changes that plastic deformation of a metallic glass leads to a distribution of free volume intermediate between a complete localization and a completely uniform distribution [49]. More recent work by Stolpe and coworkers demonstrated that based on measured shear band densities and exothermic heat releases during heating, free volume changes must have occurred throughout the metallic glass during cold rolling and not only within shear bands [50]. Recent measurements of time-dependent tracer diffusion along shear bands that showed a non-monotonic behavior with an initial increase of the diffusivity upon relaxation moreover suggest that regions in the matrix adjacent to the shear bands are affected by the deformation [51]. It is also in line with measurements of internal friction in a bulk metallic glass, where early irreversible events upon cyclic straining have been observed at strain levels lower than 0.001. In that study, it was also shown that straining the glassy material at room temperature led to a significant shift of the crystallization onset temperature, even though the crystallization occurred several tens degrees above the glass transition [52]. These results support the current results and highlight the importance of local irreversible re-configurations concerning the early stages of yielding of metallic glasses, but also stress their importance concerning the onset of crystal formation. The genesis of nanocrystals during intense deformation raises intriguing questions, for example, about the impact of deformation on quenched-in spatial heterogeneities and the indirect impact on thermally induced mechanisms such as nucleation and growth during annealing as well as concerning their possible coupling in an externally applied stress field.
